# Thalamotemporal impairment in temporal lobe epilepsy: A combined MRI analysis of structure, integrity, and connectivity

**DOI:** 10.1111/epi.12520

**Published:** 2014-01-21

**Authors:** Simon S Keller, Jonathan O'Muircheartaigh, Catherine Traynor, Karren Towgood, Gareth J Barker, Mark P Richardson

**Affiliations:** * The Department of Clinical Neuroscience, Institute of Psychiatry, King's College LondonLondon, United Kingdom; † The Department of Molecular and Clinical Pharmacology, Institute of Translational Medicine, University of LiverpoolLiverpool, United Kingdom; ‡ The Department of Neuroimaging, Institute of Psychiatry, King's College LondonLondon, United Kingdom

**Keywords:** Connectivity, Brain networks, Diffusion tensor imaging, Mesial temporal lobe, Thalamus

## Abstract

**Objective:**

Thalamic abnormality in temporal lobe epilepsy (TLE) is well known from imaging studies, but evidence is lacking regarding connectivity profiles of the thalamus and their involvement in the disease process. We used a novel multisequence magnetic resonance imaging (MRI) protocol to elucidate the relationship between mesial temporal and thalamic pathology in TLE.

**Methods:**

For 23 patients with TLE and 23 healthy controls, we performed T_1_-weighted (for analysis of tissue structure), diffusion tensor imaging (tissue connectivity), and T_1_ and T_2_ relaxation (tissue integrity) MRI across the whole brain. We used connectivity-based segmentation to determine connectivity patterns of thalamus to ipsilateral cortical regions (occipital, parietal, prefrontal, postcentral, precentral, and temporal). We subsequently determined volumes, mean tractography streamlines, and mean T_1_ and T_2_ relaxometry values for each thalamic segment preferentially connecting to a given cortical region, and of the hippocampus and entorhinal cortex.

**Results:**

As expected, patients had significant volume reduction and increased T_2_ relaxation time in ipsilateral hippocampus and entorhinal cortex. There was bilateral volume loss, mean streamline reduction, and T_2_ increase of the thalamic segment preferentially connected to temporal lobe, corresponding to anterior, dorsomedial, and pulvinar thalamic regions, with no evidence of significant change in any other thalamic segments. Left and right thalamotemporal segment volume and T_2_ were significantly correlated with volume and T_2_ of ipsilateral (epileptogenic), but not contralateral (nonepileptogenic), mesial temporal structures.

**Significance:**

These convergent and robust data indicate that thalamic abnormality in TLE is restricted to the area of the thalamus that is preferentially connected to the epileptogenic temporal lobe. The degree of thalamic pathology is related to the extent of mesial temporal lobe damage in TLE.

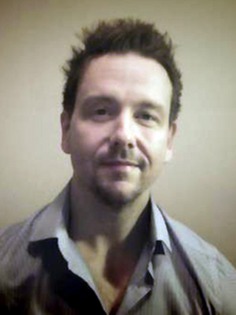



**Dr. Simon Kelle** is a lecturer and senior research scientist using advanced neuroimaging techniques in peoplewith epilepsy.

The thalamus modulates seizure activity and influences the propagation of seizures regardless of the location of the epileptogenic focus.^1^ Thalamic stimulation for the relief of intractable seizures may significantly reduce seizure frequency in patients who have not responded optimally to antiepileptic drug and/or surgical treatment.^2^,^3^ However, the mechanisms underlying reduced seizure severity after thalamic stimulation are largely unknown. Comparative animal studies of limbic epilepsy have shown that medial thalamic nuclei are important for seizure modulation and spread^4^ and show consistent neuropathologic alteration,^5^ and that dorsomedial regions have an excitatory influence on the hippocampus.^6^,^7^ Furthermore, human electrophysiologic studies, in a very small number of patients, have shown that the posterior pulvinar regions of the thalamus show ictal changes in some patients with mesial temporal seizure onset.^8^,^9^ Anterior, dorsomedial, and pulvinar regions of the thalamus preferentially connect with the temporal lobe.^10^ A localized thalamotemporal network may therefore be affected in temporal lobe epilepsy (TLE). However, evidence is lacking regarding connectivity profiles of the thalamus and their involvement in the TLE disease process. Comprehensive reviews of magnetic resonance imaging (MRI) findings in TLE have reported that the thalamus is the most affected extrahippocampal brain region in patients with TLE.^11^,^12^ One study investigated the relationship between shape changes of the thalamus and multilobar neocortical thinning in patients with TLE.^13^ Another study reported volume alterations of the thalamus, which was correlated with cortical thinning of the temporal lobe.^14^ However, all aforementioned MRI studies have solely used MRI sequences from which little information on thalamocortical connectivity can be derived (e.g., T_1_-weighted volume scans). Given (1) the opportunities to examine thalamocortical connectivity and integrity afforded by combining conventional MRI sequences with diffusion tensor imaging (DTI) and whole-brain relaxometry, and (2) the growing understanding of how network disruption may underlie seizure onset in focal and generalized epilepsies,^15^,^16^ it is increasingly important to examine the precise nature of thalamic involvement in TLE, and how thalamic alterations relate to temporal lobe abnormalities. In the present study, we recruited a sample of patients with TLE and healthy controls who each underwent an identical multisequence research MRI protocol with a primary objective of mapping alterations of thalamocortical connectivity and intrathalamic integrity in TLE, and their relationship with the disease process.

## Methods

### Participants

We recruited 23 patients with well-characterized unilateral mesial TLE (11 left-onset; mean age 40.9 years) attending King's College Hospital London and 23 healthy controls (mean age 35.3 years; no significant difference between patients and controls: p = 0.37). Diagnosis and localization of TLE was determined by comprehensive evaluation including detailed history and seizure semiology, scalp electroencephalography (EEG), intracranial EEG with video telemetry where necessary, and clinical MRI (T_1_-weighted, T_2_-weighted, and fluid-attenuated inversion recovery [FLAIR] scans). All patients had a history of complex partial seizures. In two cases, complex partial seizures were rare, and recurrent simple partial seizures were the primary seizure type. No patient had evidence of lesions other than unilateral hippocampal sclerosis diagnosed by an expert neuroradiologist. All patients had quantitative MRI evidence of unilateral hippocampal pathology ipsilateral to the side of the seizure focus determined by (1) volume loss on T_1_-weighted images, (2) increased hippocampal T_2_ values,^17^ or (3) a combination of both. Values were considered abnormal in patients if they were two standard deviations below (volume) or above (T_2_) the mean of controls. Calculation of whole hippocampal volume and mean hippocampal T_2_ values is described in the Supporting Information. Table1 provides a summary of demographic and clinical data of patients and controls. The London-Dulwich Research Ethics Committee (formerly the King's College Hospital Research Ethics Committee) approved the study, and written informed consent was obtained from all participants.

**Table 1:** Demographic and clinical data

Group	Females/males	Age	Age of onset of TLE	Duration of TLE	Seizure frequency	SGTCS
Controls	11/12	36.3 (8.1)	–	–	–	–
Left TLE	6/5	41.5 (8.3)	18.5 (14.3)	22.0 (17.2)	9.8 (10.7)	6
Right TLE	7/5	39.0 (11.7)	16.9 (13.2)	21.7 (12.3)	8.2 (8.4)	4

Data are mean (standard deviation, SD). Age, age of onset of TLE, and duration of TLE are recorded in years. Seizure frequency is number of seizures per month. SGTCS is number of patients experiencing secondary generalized tonic–clonic seizures.

### MRI acquisition

All patients and controls were scanned using an identical MR protocol on a 3 Tesla GE Signa HDx system (General Electric, Waukesha, WI, U.S.A.), with actively shielded magnetic field gradients (maximum amplitude 40 mT/m). The protocol consisted of routinely prescribed localizers, routine diagnostic sequences (two dimensional [2D] FLAIR and T_2_-weighted sequences), a three-dimensional (3D) T_1_-weighted structural volume (Inversion Recovery Spoiled Gradient Recalled echo [IR-SPGR]), a DTI sequence,^18^ and a series of 3D sequences optimized for T_1_ and T_2_ mapping (Driven Equilibrium Single Pulse Observation of T1 / T2 [DESPOT]).^19^,^20^ Details on acquisition parameters are provided in the Supporting Information.

### MRI analysis

**Figure 1:**
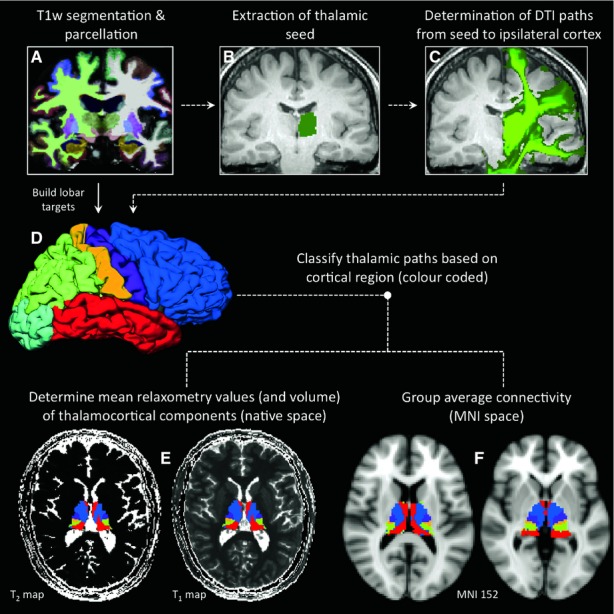
Combining T_1_-weighted, DTI, and relaxometry data to determine thalamocortical connectivity using CBS. Subcortical segmentation, gray-white matter differentiation, and regional cortical (gyral) parcellation is performed on IR-SPGR (T_1_-weighted) data using Freesurfer (A), from which thalamic seeds are extracted (B) and lobar targets are built (D). After nonlinear registration between IR-SPGR and DTI data, connectivity paths are determined from the thalamic seed to ipsilateral hemispheric regions using FSL probtrackX (C). Thalamic paths are classified according to the cortical lobar target with which they preferentially connect using “find_the_biggest” function in FSL (E; red, temporal cortex; blue, prefrontal cortex; purple, precentral cortex; yellow, postcentral cortex; green, parietal cortex; turquoise, occipital cortex). After intrasubject registration between DTI data and relaxometry maps using FMRIB's Linear Image Registration Tool (FLIRT), mean T_1_ and T_2_ values (and volumes) are calculated from each thalamocortical component (E). For group-wise average of thalamocortical segments (F), intersubject registration is applied using FMRIB's Nonlinear Image Registration Tool (FNIRT) so that data is in standard space (Montreal Neurological Institute (MNI) space; see Supporting Information).

We used connectivity-based segmentation (CBS) of the thalamus based on the original work of Behrens et al.^10^ to determine thalamocortical connectivity for each participant. Details of image analysis are provided in the Supporting Information. An overview of CBS of the thalamus is presented in Figure1. Briefly, Freesurfer software (www.Freesurfer.net, version 5.1.0) was used to obtain thalamic seeds and lobar cortical targets (occipital, parietal, prefrontal, postcentral, precentral, and temporal) for CBS analyses, and hippocampal and entorhinal regions-of-interest from each participant's IR-SPGR image. CBS of the thalamus was performed using FMRIB's (The Oxford Centre for Functional Magnetic Resonance Imaging of the Brain) Diffusion Toolbox (FDT) for probabilistic tractography running in FSL (FMRIB Software Library, http://fsl.fmrib.ox.ac.uk/fsl/fslwiki) based on DTI and IR-SPGR data. The volume of each thalamic segment preferentially connecting with a cortical target region (e.g., thalamotemporal segment, thalamoparietal segment, etc.), and of the hippocampus and entorhinal cortex, was calculated. Thalamocortical segment, hippocampal, and entorhinal volumes were analyzed, both corrected and uncorrected, for total intracranial volume (ICV); ICV was automatically obtained from Freesurfer segmentations. In order to visualize the topology of thalamocortical connectivity across individuals, thalamocortical segments were nonlinearly spatially normalized to Montreal Neurological Institute (MNI) space and averaged (see Supporting Information).

We additionally determined three further MRI measures for each structure. Mean T_1_ and T_2_ relaxation values for each thalamocortical segment, hippocampus, and entorhinal cortex was obtained by coregistering the T_1_ and T_2_ maps determined using DESPOT acquisitions with the DTI and IR-SPGR images. Furthermore, we recorded the number of streamlines between thalamus and lobar target as a proportion of the number going to all target areas. As in previous studies,^21^,^22^ the mean value for all nonzero voxels in the seed area was calculated, and was corrected for total number of streamlines to all target regions, which provided the mean proportion of streamlines to each target area. The mean proportion of streamlines provides inferential information on the degree of connectivity between thalamus and lobar target.

### Statistical analyses

Analyses were performed using SPSS (IBM Corp. Released 2012. IBM SPSS Statistics, Version 21.0.; Armonk, NY, U.S.A.). Volumetric, streamline, and relaxometry data for mesial temporal and thalamocortical segments were analyzed in each subject's native space using a multivariate analysis of variance (ANOVA) including group (control, left TLE, right TLE) as a fixed factor, and brain structure volume, mean number of streamlines, and mean T_1_ and T_2_ as dependent variables. Given the large number of comparisons (6 × thalamocortical segments, 2 × mesial temporal lobe structures, 4 × MRI measures [volume, streamlines, T_1_ and T_2_], 2 × hemispheres, 3 × groups), post hoc Bonferroni corrections were performed to resolve the direction of main effects and correct for multiple comparisons. Although there was no significant difference in mean age between patients and controls, we included age as a nuisance factor in analyses given the slightly older age of patients. Pearson's correlations were used to examine relationships between volume, mean streamlines, mean T_1_ and T_2_ values, and clinical variables. We restricted correlations between brain regions to values from the same MRI sequence (i.e., volume with volume, T_1_ with T_1_, and T_2_ with T_2_). Given that mean streamlines are obtained only for each thalamocortical segment, streamlines were only correlated with clinical variables and not mesial temporal values from other sequences.

## Results

All main effects and F-values are tabulated in the Supporting Information.

### Medial temporal lobe

There were significant main effects of left entorhinal volume (F = 11.09, p < 0.001) and T_2_ (F = 9.15, p = 0.001), right entorhinal volume (F = 6.05, p = 0.005) and T_2_ (F = 8.51, p = 0.001), left hippocampal volume (F = 9.43, p < 0.001), T_1_ (F = 4.90, p = 0.01), and T_2_ (F = 7.39, p = 0.002), and right hippocampal volume (F = 10.12, p < 0.001) and T_2_ (F = 3.49, p = 0.04). Post hoc Bonferroni corrected results (Table2) indicated decreased volume of the ipsilateral entorhinal cortex (left TLE: p < 0.001; right TLE: p = 0.005) and hippocampus (left TLE: p < 0.001; right TLE: p < 0.001) in patients relative to controls. These findings were also obtained when corrected for ICV. Mean T_2_ values were significantly increased in the ipsilateral entorhinal cortex (left TLE: p < 0.001; right TLE p < 0.001) and hippocampus (left TLE: p = 0.002; right TLE: p = 0.045) in patients relative to controls. There was also a significant increase in mean T_2_ of the contralateral entorhinal cortex in patients with left TLE relative to controls (p = 0.02).

**Table 2:** Post hoc Bonferroni-corrected differences in mesial temporal lobe regions between patients and controls.

Region	Hemisphere	Measure	Comparison	Bonferroni Sig.
Entorhinal	L	Volume	Ctrl – L TLE Ctrl – R TLE	0.000***0.033*
				T_1_	Ctrl – L TLE Ctrl – R TLE	0.147 1.00
				T_2_	Ctrl – L TLE Ctrl – R TLE	0.000*** 0.560
		R	Volume	Ctrl – L TLE Ctrl – R TLE	1.00 0.005**
				T_1_	Ctrl – L TLE Ctrl – R TLE	0.374 1.00
				T_2_	Ctrl – L TLE Ctrl – R TLE	0.020* 0.000***
Hippocampus	L	Volume	Ctrl – L TLE Ctrl – R TLE	0.000*** 1.00
				T_1_	Ctrl – L TLE Ctrl – R TLE	0.035*1.00
				T_2_	Ctrl – L TLE Ctrl – R TLE	0.002** 1.00
		R	Volume	Ctrl – L TLE Ctrl – R TLE	1.00 0.000***
				T_1_	Ctrl – L TLE Ctrl – R TLE	0.542 0.214
				T_2_	Ctrl – L TLE Ctrl – R TLE	0.409 0.045*

*** p < 0.01

** p < 0.01

* p < 0.05.

Main effects and corresponding F values are provided in the Supporting Information

### Connectivity-based thalamic segmentation

**Figure 2:**
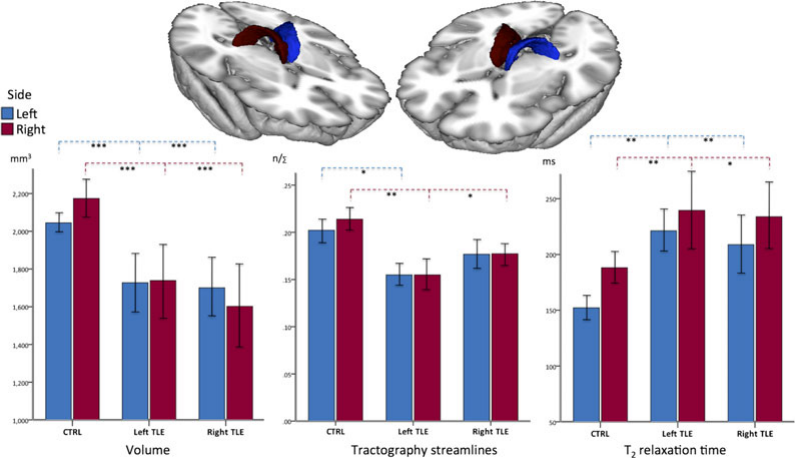
Differences in volume, proportional number of tractography streamlines, and T_2_ relaxation of the ipsilateral and contralateral thalamotemporal segments in patients relative to controls. Top: Three-dimensional rendering of the left (blue) and right (red) thalamotemporal segments averaged from all subjects in MNI space (see Supporting Information). Bottom left: volume reduction in patients relative to controls. Bottom middle: corrected mean number of tractography streamlines reduced in patients relative to controls. Bottom right: T_2_ relaxation increase in patients relative to controls. Blue and red bars represent the left and right thalamotemporal segments, respectively. ***p < 0.001; **p < 0.01; *p < 0.05.

There were significant main effects of the volume of the left thalamoparietal segment (F = 4.03, p = 0.03), thalamopostcentral segment (F = 3.51, p = 0.04), thalamotemporal segment (F = 21.52, p < 0.001), whole thalamus (F = 6.53, p = 0.003), and right thalamotemporal segment (F = 17.96, p < 0.001). The remaining significant main effects were restricted to left and right thalamotemporal mean T_2_ (F = 14.99, p < 0.001 and F = 5.53, p = 0.007, respectively) and left and right thalamotemporal mean number of streamlines (F = 5.27, p = 0.009 and F = 7.43, p = 0.002, respectively). Table3 presents the Bonferroni corrected results, and indicates that other than significantly reduced ipsilateral whole thalamic volume in patients with left TLE relative to controls (p = 0.002), all significant differences in volume, mean T_2_ and mean number of streamlines between patients and controls were restricted to the left and right thalamotemporal segments. This included reduction in ipsilateral (left TLE p < 0.001, right TLE p < 0.001) and contralateral (left TLE p = 0.001, right TLE p < 0.001) volume, increased ipsilateral (left TLE p < 0.001, right TLE p = 0.04) and contralateral (left TLE p = 0.02, right TLE p < 0.001) mean T_2_, and reduced ipsilateral (left TLE p = 0.03, right TLE p = 0.04) and contralateral (left TLE only p = 0.001) mean number of streamlines, in patients relative to controls. These combined thalamotemporal alterations are illustrated in Figure2. Although not achieving statistical significance, there were nonspecific increases in the corrected number of streamlines and volume of other thalamocortical segments in patients relative to controls. In particular, there was a strong trend for an increased number of streamlines within the thalamoprecentral segment in patients relative to controls (p = 0.07, Table3). Significant reduction of thalamotemporal segment volume in patients was also seen when corrected for ICV and whole thalamic volume. The spatial organization of the averaged thalamocortical segments across individuals can be visualized in Figure1F and in more detail in the Supporting Information. In particular, the thalamotemporal segment that showed the only differences in structure, integrity, and connectivity in patients was located in anterior, dorsomedial, and posterior thalamic regions.

**Table 3:** Post hoc Bonferroni-corrected differences in thalamocortical segments between patients and controls.

Region	Hemisphere	Measure	Comparison	Bonferroni Sig.
Thalamus: occipital	L	Volume	Ctrl – L TLE Ctrl – R TLE	0.471 1.00
				T_1_	Ctrl – L TLE Ctrl – R TLE	1.00 1.00
				T_2_	Ctrl – L TLE Ctrl – R TLE	1.00 1.00
				Streamlines	Ctrl – L TLE Ctrl – R TLE	0.355 1.00
		R	Volume	Ctrl – L TLE Ctrl – R TLE	1.00 0.872
				T_1_	Ctrl – L TLE Ctrl – R TLE	0.079 0.533
				T_2_	Ctrl – L TLE Ctrl – R TLE	1.00 1.00
				Streamlines	Ctrl – L TLE Ctrl – R TLE	0.447 1.00
Thalamus: parietal	L	Volume	Ctrl – L TLE Ctrl – R TLE	0.058 0.337
				T_1_	Ctrl – L TLE Ctrl – R TLE	1.00 1.00
				T_2_	Ctrl – L TLE Ctrl – R TLE	1.00 1.00
				Streamlines	Ctrl – L TLE Ctrl – R TLE	0.534 1.00
		R	Volume	Ctrl – L TLE Ctrl – R TLE	0.708 0.332
				T_1_	Ctrl – L TLE Ctrl – R TLE	1.00 0.690
				T_2_	Ctrl – L TLE Ctrl – R TLE	1.00 1.00
				Streamlines	Ctrl – L TLE Ctrl – R TLE	1.00 1.00
Thalamus: PFC	L	Volume	Ctrl – L TLE Ctrl – R TLE	1.00 1.00
				T_1_	Ctrl – L TLE Ctrl – R TLE	1.00 1.00
				T_2_	Ctrl – L TLE Ctrl – R TLE	1.00 1.00
				Streamlines	Ctrl – L TLE Ctrl – R TLE	1.00 1.00
		R	Volume	Ctrl – L TLE Ctrl – R TLE	1.00 1.00
				T_1_	Ctrl – L TLE Ctrl – R TLE	1.00 5.13
				T_2_	Ctrl – L TLE Ctrl – R TLE	1.00 1.00
				Streamlines	Ctrl – L TLE Ctrl – R TLE	0.45 0.98
Thalamus: postcentral	L	Volume	Ctrl – L TLE Ctrl – R TLE	0.124 0.100
				T_1_	Ctrl – L TLE Ctrl – R TLE	1.00 1.00
				T_2_	Ctrl – L TLE Ctrl – R TLE	1.00 1.00
				Streamlines	Ctrl – L TLE Ctrl – R TLE	1.00 1.00
		R	Volume	Ctrl – L TLE Ctrl – R TLE	1.00 1.00
				T_1_	Ctrl – L TLE Ctrl – R TLE	0.487 1.00
				T_2_	Ctrl – L TLE Ctrl – R TLE	1.00 0.722
				Streamlines	Ctrl – L TLE Ctrl – R TLE	0.42 0.65
Thalamus: precentral	L	Volume	Ctrl – L TLE Ctrl – R TLE	1.00 1.00
				T_1_	Ctrl – L TLE Ctrl – R TLE	0.985 1.00
				T_2_	Ctrl – L TLE Ctrl – R TLE	1.00 0.834
				Streamlines	Ctrl – L TLE Ctrl – R TLE	1.00 1.00
		R	Volume	Ctrl – L TLE Ctrl – R TLE	1.00 0.692
				T_1_	Ctrl – L TLE Ctrl – R TLE	1.00 1.00
				T_2_	Ctrl – L TLE Ctrl – R TLE	1.00 1.00
				Streamlines	Ctrl – L TLE Ctrl – R TLE	0.07 0.259
Thalamus: temporal	L	Volume	Ctrl – L TLE Ctrl – R TLE	0.000*** 0.000***
				T_1_	Ctrl – L TLE Ctrl – R TLE	1.00 1.00
				T_2_	Ctrl – L TLE Ctrl – R TLE	0.000*** 0.000***
				Streamlines	Ctrl – L TLE Ctrl – R TLE	0.034* 0.304
		R	Volume	Ctrl – L TLE Ctrl – R TLE	0.001** 0.000***
				T_1_	Ctrl – L TLE Ctrl – R TLE	1.00 0.227
				T_2_	Ctrl – L TLE Ctrl – R TLE	0.024* 0.038*
				Streamlines	Ctrl – L TLE Ctrl – R TLE	0.001** 0.041*
Thalamus: whole	L	Volume	Ctrl – L TLE Ctrl – R TLE	0.002** 0.114
				T_1_	Ctrl – L TLE Ctrl – R TLE	0.772 1.00
				T_2_	Ctrl – L TLE Ctrl – R TLE	0.243 1.00
		Streamlines	Ctrl – L TLE Ctrl – R TLE	– –
		R	Volume	Ctrl – L TLE Ctrl – R TLE	0.173 0.231
				T_1_	Ctrl – L TLE Ctrl – R TLE	1.00 0.174
				T_2_	Ctrl – L TLE Ctrl – R TLE	1.00 0.413
				Streamlines	Ctrl – L TLE Ctrl – R TLE	– –

*** p < 0.001

** p < 0.01

* p < 0.05.

Main effects and corresponding F values are provided in the Supporting Information. Statistically significant differences between patients and controls are restricted to thalamotemporal segment volume, T_2_ and streamlines, with no significant effects in any other segment. Ipsilateral whole thalamic volume is also significantly reduced in patients with left TLE. Analysis of thalamocortical segment streamlines are performed corrected for total number of streamlines

### Correlations and clinical variables

**Figure 3:**
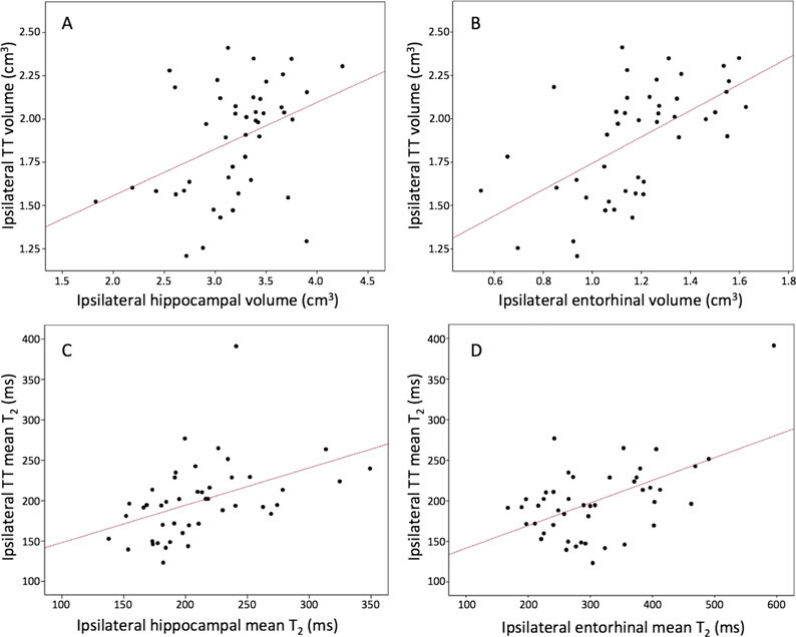
Significant correlations between ipsilateral mesial temporal and thalamotemporal (TT) structures. (A) hippocampal and TT volume; (B) entorhinal and TT volume; (C) hippocampal and TT mean T_2_; (D) entorhinal and TT mean T_2_. Correlations were also observed between the ipsilateral mesial temporal and contralateral TT structures (not pictured, see main text), but not with contralateral mesial temporal structures.

Given that significant differences between patients and controls were limited to mesial temporal structures and thalamotemporal segments, we restricted correlation analyses to these regions. Furthermore, because patients with left and right TLE showed similar mesial temporal and thalamotemporal segment alterations, we combined patients with left- and right-sided TLE into one sample for correlational analyses to increase statistical sensitivity, and treated structures as ipsilateral and contralateral to the epileptogenic focus. Correlational analyses indicated that ipsilateral hippocampal and entorhinal volume were significantly correlated with ipsilateral (r = 0.39, p = 0.008 and r = 0.58, p < 0.001, respectively) and contralateral (r = 0.39, p = 0.02 and r = 0.52, p < 0.001, respectively) thalamotemporal segment volume. Similarly, ipsilateral hippocampal and entorhinal mean T_2_ values were correlated with ipsilateral (r = 0.45, p = 0.002 and r = 0.55, p < 0.001, respectively) and contralateral (r = 0.63, p < 0.001 and r = 0.35, p < 0.02, respectively) thalamotemporal segment mean T_2_. Ipsilateral mesial temporal and thalamotemporal correlations are shown in Figure3. There were no correlations with contralateral (i.e., nonepileptogenic) mesial temporal structures. Ipsilateral thalamocortical segment mean T_2_ was correlated with duration of epilepsy (number of years; r = 0.48, p = 0.02) and seizure frequency (number of complex partial seizures per year; r = 0.45, p = 0.03). Ipsilateral thalamocortical segment mean number of streamlines was also correlated with seizure frequency (r = −0.44, p = 0.03). There were no other statistically significant relationships.

## Discussion

The thalamus is known to be a crucial component of the seizure-generating network in generalized spike-wave, which is characteristic of idiopathic (or genetic) generalized epilepsy (IGE).^23^ Global structural impairments (e.g., volume loss) of the thalamus have been reported previously in IGE.^24^,^25^ Prior evidence strongly suggests that thalamus is affected in epilepsies with focal-onset seizures, possibly as part of a seizure-generating network,^26^ although specific details of the involvement of thalamus in human TLE are lacking. In TLE, thalamic involvement has typically been reported in terms of nonspecific global volume loss,^1^,^12^,^14^ or nonspecific DTI-determined diffusion abnormalities.^27^,^28^ However, the thalamus has a complex internal structure, sending and receiving connections to different cortical regions across the entire brain from different regions within the thalamus. In the present study we report for the first time that the specific regions of the thalamus that were demonstrated to preferentially connect with the temporal lobe are abnormal bilaterally in TLE, and that the severity of abnormality is correlated with the degree of mesial temporal damage in the epileptogenic temporal lobe. Regions of the thalamus that were demonstrated to be preferentially connected with other brain regions showed no pathologic change.

The organization of thalamocortical connections in this paper is consistent with the work of Behrens et al.^10^ in healthy controls, indicating that the temporal lobe preferentially connects with anterior, dorsomedial, and posterior regions of the thalamus (see Fig.1F and Supporting Information), corresponding to the anterior nuclear group, the dorsomedial nucleus, and the pulvinar, respectively. It is these thalamic nuclear regions that are abnormal in terms of structure, connectivity (as suggested in analysis of volume and DTI streamlines) and integrity (T_2_ relaxometry) in patients with TLE. Our data are consistent with, build on, and integrate previous studies that report

1. that dorsomedial thalamic regions have an excitatory influence on the hippocampus in animal models;^6^,^7^
2. neuronal loss and physiologic alterations in the dorsomedial thalamus in animal models of limbic epilepsy;^4^,^5^
3. shape alterations of the thalamus in regions hypothesized—but not demonstrated—to be connected to the temporal lobe in TLE;^13^
4. preferential gray matter loss of a region corresponding to the dorsomedial area of the thalamus in a recent review of voxel-based morphometry studies of TLE;^11^
5. 2-deoxy-2-[^18^F]fluoro-d-glucose and [^11^C]flumazenil positron emission tomography (PET) abnormalities of the dorsomedial thalamic region in patients with TLE;^29^
6. ictal changes in the pulvinar during mesial temporal lobe seizures based on stereoelectroencephalographic (SEEG) data in a small number of cases.^8^,^9^

Despite the fact that structural and metabolic alterations of the medial temporal lobe^12^,^30^ and thalamus^1^,^12^,^30^ are frequently reported in TLE, there are few studies examining altered connectivity between the temporal lobe and thalamus in these patients, which would provide more direct evidence of an epileptogenic network. Correlating neural dysfunction^30^ and measures of atrophy^13^,^14^ between thalamus and temporal lobe has been suggestive of thalamotemporal network pathology, but there have been no attempts to identify regional thalamic alterations based on the determined cortical connectivity patterns of the thalamus, using multiple measures of tissue alteration. Mueller et al.^14^ and Bonilha et al.^31^ provided evidence of region-specific atrophy of the anterior thalamus in TLE, while Bernhardt et al.^13^ reported atrophy of anterior, medial, and posterior thalamus. Despite that the aforementioned authors describe alterations of the thalamus in context of its connectivity with the temporal lobe, all evidence of thalamic structural alteration was derived from T_1_-weighted MRI scans only, which provides very little—if any—information on brain connectivity. Our data are more consistent with that of Bernhardt et al.^13^ in terms of the spatial topology of thalamic abnormality, but we delineate the thalamic region preferentially connecting to the temporal lobe, and provide combined measures of region-specific thalamic pathology based on a multisequence MRI approach.

The etiology of thalamotemporal abnormalities in TLE is difficult to resolve. Competing hypotheses of excitotoxic damage and deafferentation have been proposed to explain extrahippocampal atrophy in TLE, and it is possible that both mechanisms play a role in thalamic damage. Our finding of correlations between the extent of ipsilateral mesial temporal and thalamotemporal segment damage are somewhat consistent with that of Mueller et al.,^14^ who reported a relationship between volume loss of the thalamus and cortical thinning of the mesial temporal lobe, and interpreted these results as suggestive of progressive thalamic degeneration due to seizure propagation from the connected epileptogenic temporal lobe. Barron et al.^11^ also suggested that thalamic damage could result from mesial temporal excitotoxicity, given that that the epileptogenic hippocampus and dorsomedial area of the thalamus are nodes within the same network, thereby providing a route for excitotoxic damage. These interpretations are partially supported by our data that indicate that the degree of the thalamic damage is correlated with the extent of damage to the epileptogenic mesial temporal lobe but not the contralateral side. In conjunction with commissural and brainstem mediated spread, the thalamus serves as an interhemispheric ictal propagation pathway,^32^ which may in part explain the bilateral thalamotemporal damage observed in this study. Furthermore, the extent of thalamic damage appears to be related to duration of epilepsy (as indicated in this study and in others^27^,^33^), which may be suggestive of a degenerative effect on the thalamus due to the chronicity of TLE.^34^

On the other hand, Bonilha et al.^35^ suggested that the excitotoxic effects of seizures would be reduced when the epileptogenic zone becomes increasingly disconnected from other cortical and subcortical regions, and that such disconnection may also contribute to neuronal damage in some brain regions. Our results are also in support of this, given that a circumscribed loss of thalamotemporal segment volume and reduction of mean streamlines is suggestive of reduced structural connectivity between the temporal lobe and thalamus. Thalamotemporal deafferentation is a plausible hypothesis in light of findings from a recent computer simulation study of EEG data that indicated that decreasing connectivity between brain regions increases the frequency of seizure-like activity.^16^ This seemingly counterintuitive finding may be explained by the notion of fewer connections creating a less stable network, which may facilitate the transition to ictal dynamics.^15^ There has been a recent move to understand how seizures begin and terminate through the study of macrostructural networks using animal models, neuroimaging data, and simulated data;^16^ loss of connectivity between brain regions is a legitimate hypothesis for the etiology of both focal and generalized seizures. This new direction of research mirrors recent modifications in the classification of epilepsy disorders to consider the importance of brain networks for seizure onset, even in focal epilepsies.^36^,^37^ Our findings of reduced thalamotemporal connectivity in patients with TLE are consistent with other data indicating loss of general temporal lobe connectivity,^38^ and together suggest that the phenomenologic concept of network disruption causing seizures has an anatomic basis.

It is important to highlight pertinent methodologic issues. Firstly, we present data on a sample size smaller than many structural MRI studies of TLE, including work from some members of our research team.^27^,^33^,^39^ It is likely that the majority of previous structural MRI work in TLE has been performed in context of routine clinical/presurgical evaluation, and has benefited from routinely acquired MRIs. Here, we prospectively recruited patients and controls for a multisequence MRI research protocol specifically to investigate how the brain is altered in terms of structure, integrity, and connectivity in TLE, which is typically beyond the scope of clinical MRI protocols. Despite the relatively small sample size, we present very robust statistical evidence of circumscribed thalamotemporal pathology in TLE. Secondly, CBS and DTI are not without limitations. An in-depth critique of DTI is beyond the scope of this paper, and the reader is referred to an excellent recent discussion by Jones et al.^40^ that covers a wide and detailed range of issues and considerations for all DTI research in clinical populations. Of particular relevance to the present study are the difficulties in modelling crossing fibers within a single voxel, the relatively low isotropic voxel size used, and the recommendations for increased number of gradient orientations. Furthermore, CBS only provides a relative value of thalamocortical segment connectivity; where we observed reduced connectivity streamlines in thalamotemporal segments in patients, there were nonspecific concomitant increases in other thalamocortical segments. Given that segments with fewer connectivity streamlines will always be accompanied by increased streamlines in other ipsilateral thalamic segments, such is the nature of the technique, it is wise to exercise caution when interpreting intrathalamic reorganization using this method. However, our finding of selective involvement of the thalamotemporal segment is reinforced by the circumscribed increase in T_2_, the calculation of which is not dependent on the CBS technique.

## Conclusion

Thalamic abnormality in TLE is restricted to the area of the thalamus that is preferentially connected with the epileptogenic temporal lobe. Although it is impossible to determine the directionality of any influences between brain regions using these neuroimaging data (i.e., do mesial temporal seizures cause damage to specific connected thalamic regions, does the disease process in specific thalamic regions permit increasingly degenerative changes of the epileptogenic temporal lobe, or are the two structures affected simultaneously), we do provide convincing evidence of a relationship between the degree of circumscribed thalamotemporal segment pathology and the extent of abnormality within the epileptogenic mesial temporal lobe.

## Supporting Information

Data S1 Methods and results.
