# Supplementary material for: Thalamotemporal impairment in temporal lobe epilepsy: A combined MRI analysis of structure, integrity, and connectivity
Source: Epilepsia. 2014 Jan 21;55(2):306–15. doi: 10.1111/epi.12520 (PMC4074767; doi:10.1111/epi.12520)
Supplement: Data S1 — Methods and results. [file epi0055-0306-SD1.docx]

Supplementary materials

**Thalamotemporal impairment in temporal lobe epilepsy:**

**A combined MRI analysis of structure, integrity and connectivity**

Simon S. Keller^1,3^, Jonathan O’Muircheartaigh^2^, Catherine Traynor^1^,

Karren Towgood^1^, Gareth J. Barker^2^, Mark P. Richardson^1^

The Departments of ^1^Clinical Neuroscience and ^2^Neuroimaging, Institute of Psychiatry,

King’s College London, London SE5 8AF, UK

^3^The Department of Molecular and Clinical Pharmacology, Institute of Translational Medicine, University of Liverpool, UK

**Supplementary Methods**

***Image acquisition***

The 3D T_1_-weighted scan was a coronal inversion recovery prepared spoiled gradient-echo (IR-SPGR) sequence with an acquisition time of approximately 6 minutes (TR=7.2 ms, TE=2.8 ms, TI=450 ms, flip angle=20, NEX=0.75, slice thickness=1.1 mm, in plane field of view 28.2cm, 256 x 256 x 160 matrix). DTI acquisition was derived from the protocol described by Jones et al.[^1^](#_ENREF_1) Each volume of DTI data was acquired using a cardiac-gated, multi-slice peripherally-gated doubly refocused spin echo echo-planar imaging (EPI) sequence, optimized for precise measurement of the diffusion tensor in parenchyma, from 60 contiguous near-axial slice locations with isotropic (2.4 x 2.4 x 2.4 mm) voxels. The echo time was 104.5 ms while the effective repetition time varied between subjects in the range 12 and 20 RR intervals and an ASSET speed up factor of 2 was used. Thirty-two images were collected at a diffusion weighting of 1300 s mm**^−^**^2^ **-** with gradient directions uniformly distributed in space **-** together with four images acquired with no diffusion gradients applied. T_1_ and T_2_ maps were acquired using the Driven Equilibrium Single Pulse Observation of T_1_ & T_2_ (DESPOT) method described by Deoni et al.[^2^](#_ENREF_2)^,^ [^3^](#_ENREF_3) To generate the T_1_ map, two spoiled gradient echo (SPGR) images (TR=7.93 ms, TE=3.75 ms, excitation flip angles 18° and 4°) and one inversion recovery prepared SPGR (IR-SPGR) image (TR=7.68 ms, TE=3.75 ms, TI=450 ms, flip angle 5°) were acquired. For the T_2_ maps, five steady state free precession (SSFP) images were acquired using different combinations of flip angle and phase cycling schemes (TR=4.5 ms, excitation flip angles 65°, and 15° each with phase=0° and excitation flip angles 65° and 15° with phase=180°, 256 × 256 × 160 matrix). For all images (except for the IR-SPGR, for which a large voxel size was used), the in-plane FOV was 28 cm and slice thickness was 1.1 mm, resulting in a voxel size of 1.1 × 1.1 × 1.1 mm^3^. The entire scanning protocol lasted approximately 45 minutes per participant.

***Image analysis***

***Brain parcellation.*** To obtain automated, observer-independent, thalamic seeds and cortical targets for DTI analyses, and regions-of-interest for volume and relaxometry analyses, the FreeSurfer software package (<http://surfer.nmr.mgh.harvard.edu>, version 5.1.0) was applied to the IR-SPGR images (Fig 1a). Detailed descriptions of cortical reconstruction, subcortical labelling and volume estimation using Freesurfer are provided elsewhere.[^4^](#_ENREF_4)^,^ [^5^](#_ENREF_5) Briefly, each IR-SPGR image was submitted to the ‘recon-all’ processing stream, which, amongst other functions, automatically segments the cortical ribbon, parcellates individual sulci and gyri, and labels individual subcortical structures. In the present study, cortical parcellations and thalamic labels were converted to individual masks that could be used for connectivity-based segmentation (CBS) using both Freesurfer functions and routines from FSL (the fMRIB Library, version 4.2.0; http://fsl.fmrib.ox.ac.uk/fsl/fslwiki/) functions. Left and right thalamic masks (Fig 1b) were generated by converting parcellated Freesurfer image files to NIfTI format, and extracting the thalamic labels. In order to determine masks for cortical targets for CBS (Fig 1d), we first converted cortical parcellated annotation files to individual labels. This generated multiple gyral labels from the Desikan-Killiany atlas,[^6^](#_ENREF_6) from which lobar labels were created (Supplementary Table 1). Lobar labels were subsequently converted into binarised NIfTI files, which provided cortical classification targets for CBS analyses. Hippocampal and entorhinal masks were also extracted for each participant using FreeSurfer software and the same extraction methods as the thalamus. The spatial location of entorhinal cortex was determined based on the work of Fischl et al.,[^7^](#_ENREF_7) which delineates the entorhinal region based on cortical folding patterns (Supplementary Figure 1).

| **Structure** | **Anatomical Labels** |
| --- | --- |
| Occipital cortex | Cuneus, lateral occipital, lingual, pericalcarine |
| Parietal cortex | Inferior parietal, precuneus, superior parietal, supramarginal |
| Prefrontal cortex | Caudal middle frontal, lateral orbitofrontal, medial orbitofrontal, pars opercularis, pars triangularis, pars orbitalis, rostral middle frontal, superior frontal, frontal pole |
| Postcentral cortex | Postcentral |
| Precentral cortex | Precentral, paracentral |
| Temporal cortex | Superior temporal sulcus, entorhinal, fusiform, inferior temporal, middle temporal, parahippocampal, superior temporal, temporal pole, transverse temporal |

**Supplementary Table 1.** Merging of Freesurfer anatomical label parcellations to form cortical classification targets for CBS analyses.

**Supplementary Figure 1.** Location of entorhinal cortex in two randomly selected patients with TLE.
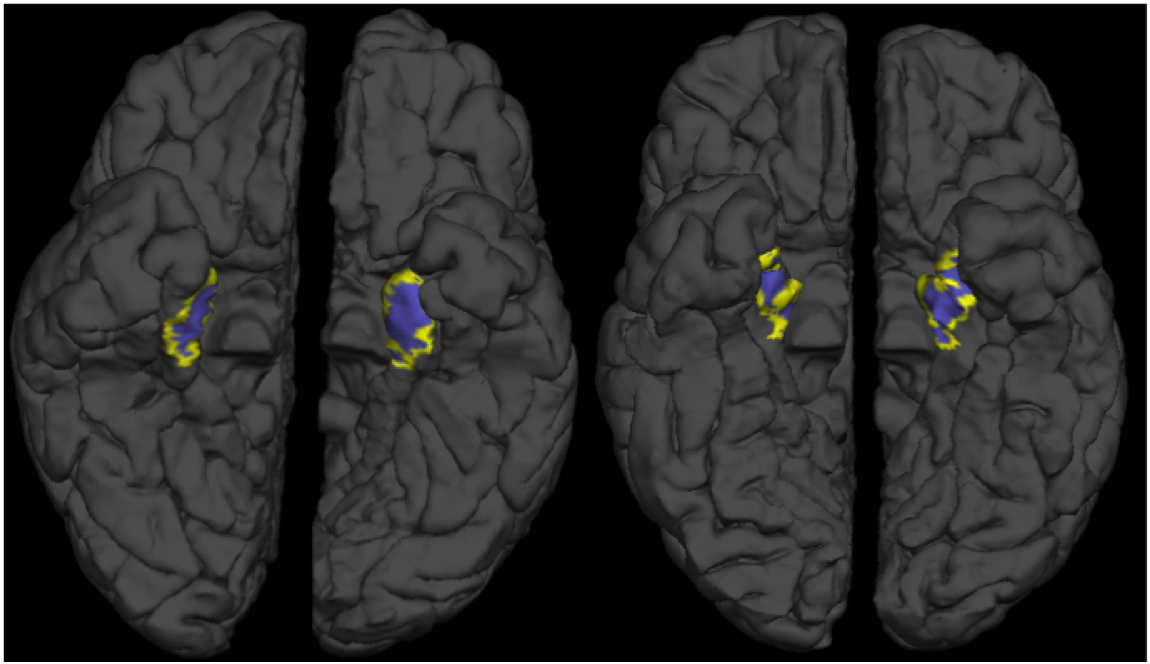


***Connectivity-based segmentation (CBS).*** CBS of the thalamus was based on the original work of Behrens et al.[^8^](#_ENREF_8) using FMRIB’s Diffusion Toolbox (FDT) for probabilistic tractography running in FSL. This method has been shown to be highly reproducible within and between subjects for segmentation of the thalamus according to lobar cortical connectivity.[^9^](#_ENREF_9) Prior to probabilistic tracking of fibres, DTI data were pre-processed by (i) correcting for the effects of eddy currents, (ii) brain extracting a non-diffusion weighted volume using the Brain Extraction Tool (BET), (iii) reconstructing the diffusion tensors using DTIFIT, (iv) determining the diffusion parameters at each voxel, using ‘bedpostX’ and (v) generating transformation matrices from non-linearly registered IR-SPGR and DTI data in native space. Following preprocessing, ‘probtrackX’ was used for probabilistic tractography using the thalamic seed and cortical termination masks generated by Freesurfer from the IR-SPGR data. Processing was carried out independently for each cerebral hemisphere, with connections from each thalamus to the ipsilateral cortical target regions being investigated (main Figure 1c, d). One benefit of using Freesurfer for the definition of target cortical regions is the excellent delineation of cortex from underlying white matter for the definition of cortical targets, which has sub-millimetre accuracy.[^10^](#_ENREF_10) Grey matter only cortical target regions are necessary to ensure that tractography paths stop as soon as they enter the cortex, and to prevent paths from passing from one target region into another. We were interested in only ipsilateral connections, so streamlines to the contralateral hemisphere were masked out (main Figure 1c). For each target region, the number of connections from voxels in the thalamus to the target cortical region was found. This information was output as an image for each target region. The outputs produced for each target region were then combined using the “find_the_biggest” utility in FSL, which classified each voxel depending on the target to which the greatest number of the paths from that voxel propagated (main Figure 1e). This process was a “hard segmentation”, in which a single label was assigned to each thalamic voxel. The volume of each thalamocortical segment (thalamotemporal, thalamoparietal, etc) and hippocampal and entorhinal mask was calculated using ‘fslstats’.

To visualise the common thalamocortical connectivity profiles for each subject group, thalamocortical segments were spatially transformed into stereotaxic (Montreal Neurological Institute, MNI) space. This was performed by first determining non-linear transformation matrices between T1-weighted native space and standardised space (default MNI152 T1-weighted template in FSL) using FSL FNIRT. Non-linear warps obtained from these matrices were subsequently applied with nearest neighbour interpolation to the individual thalamocortical parcellations for each subject, which transformed parcellations into MNI space. Within-subject thalamocortical segments were separated using ‘fslmaths’, between-subject mean maps of individual thalamocortical segments were calculated using ‘fslmerge’ and ‘fslmaths’, and hard segmentations of group-wise thalamocortical segments was performed using the ‘find_the_biggest’ function.

***T_1_ and T_2_ relaxometry maps.*** To calculate T_1_ and T_2_ maps, images from the DESPOT acquisition first had to be co-registered. The DESPOT image acquired using the highest flip angle (18°) was used as the reference image. All other images from the DESPOT sequences were registered to this image using FSL FLIRT. For all registration steps, a 12-degree of freedom affine transformation was used, with the mutual information cost function and tri-linear interpolation. Following this, T_1_ maps were generated using DESPOT-HIFI[^2^](#_ENREF_2) and T_2_ maps were obtained using DESPOT-FM[^3^](#_ENREF_3) running in ImageJ (<http://rsbweb.nih.gov/ij/>). T_1_ and T_2_ maps were subsequently registered to the IR-SPGR structural images from which thalamic seeds and cortical targets were obtained (and with which DTI data was also spatially registered) using FLIRT and nearest neighbour interpolation. This provided co-localisation of the thalamocortical segments obtained from probabilistic tractography - and hippocampal and entorhinal regions - to relaxometry maps, from which mean T_1_ and T_2_ values were extracted using ‘fslstats’.

**Supplementary Results**

***Mesial temporal lobe main effects***

| **Region / measure** | **F** | **Sig.** |
| --- | --- | --- |
| Left entorhinal: volume | 11.086 | .000 |
| Left entorhinal: T_1_ | 3.109 | .055 |
| Left entorhinal: T_2_ | 9.150 | .001 |
| Right entorhinal: volume | 6.048 | .005 |
| Right entorhinal: T_1_ | 1.274 | .290 |
| Right entorhinal: T_2_ | 8.512 | .001 |
| Left hippocampal: volume | 9.432 | .000 |
| Left hippocampal: T_1_ | 4.903 | .012 |
| Left hippocampal: T_2_ | 7.391 | .002 |
| Right hippocampal: volume | 10.119 | .000 |
| Right hippocampal: T_1_ | 2.038 | .143 |
| Right hippocampal: T_2_ | 3.490 | .040 |

***Connectivity-based thalamic segmentation main effects***

| **Measure** | **Region** | **F** | **Sig.** |
| --- | --- | --- | --- |
| Volume | Left thalamus: occipital | 1.406 | .256 |
|  | Left thalamus: parietal | 4.034 | .025 |
|  | Left thalamus: pfc | .923 | .405 |
|  | Left thalamus: postcentral | 3.510 | .039 |
|  | Left thalamus: precentral | .620 | .543 |
|  | Left thalamus: temporal | 21.521 | .000 |
|  | Left thalamus: global | 6.529 | .003 |
|  | Right thalamus: occipital | .636 | .534 |
|  | Right thalamus: parietal | 1.584 | .217 |
|  | Right thalamus: pfc | .098 | .906 |
|  | Right thalamus: postcentral | .463 | .632 |
|  | Right thalamus: precentral | .978 | .385 |
|  | Right thalamus: temporal | 17.961 | .000 |
|  | Right thalamus: global | 2.684 | .080 |

| **Measure** | **Region** | **F** | **Sig.** |
| --- | --- | --- | --- |
| T_1_ | Left thalamus: occipital | .192 | .826 |
|  | Left thalamus: parietal | .159 | .854 |
|  | Left thalamus: pfc | .160 | .852 |
|  | Left thalamus: postcentral | .346 | .709 |
|  | Left thalamus: precentral | .524 | .596 |
|  | Left thalamus: temporal | .387 | .681 |
|  | Left thalamus: global | .661 | .522 |
|  | Right thalamus: occipital | 2.826 | .070 |
|  | Right thalamus: parietal | 1.167 | .321 |
|  | Right thalamus: pfc | 1.442 | .248 |
|  | Right thalamus: postcentral | 1.030 | .366 |
|  | Right thalamus: precentral | .238 | .789 |
|  | Right thalamus: temporal | 2.317 | .111 |
|  | Right thalamus: global | 2.802 | .072 |

| **Measure** | **Region** | **F** | **Sig.** |
| --- | --- | --- | --- |
| T_2_ | Left thalamus: occipital | .176 | .839 |
|  | Left thalamus: parietal | .167 | .847 |
|  | Left thalamus: pfc | .333 | .719 |
|  | Left thalamus: postcentral | .355 | .703 |
|  | Left thalamus: precentral | .644 | .530 |
|  | Left thalamus: temporal | 14.992 | .000 |
|  | Left thalamus: global | 1.608 | .212 |
|  | Right thalamus: occipital | 4.086 | .024 |
|  | Right thalamus: parietal | .322 | .727 |
|  | Right thalamus: pfc | .568 | .571 |
|  | Right thalamus: postcentral | 1.088 | .346 |
|  | Right thalamus: precentral | .337 | .716 |
|  | Right thalamus: temporal | 5.528 | .007 |
|  | Right thalamus: global | 1.669 | .201 |

| **Measure** | **Region** | **F** | **Sig.** |
| --- | --- | --- | --- |
| Streamlines | Left thalamus: occipital | 1.337 | .274 |
|  | Left thalamus: parietal | .956 | .393 |
|  | Left thalamus: pfc | .157 | .855 |
|  | Left thalamus: postcentral | .061 | .941 |
|  | Left thalamus: precentral | .456 | .637 |
|  | Left thalamus: temporal | 3.693 | .021 |
|  | Left thalamus: global | - | - |
|  | Right thalamus: occipital | 1.083 | .348 |
|  | Right thalamus: parietal | .473 | .626 |
|  | Right thalamus: pfc | 1.221 | .305 |
|  | Right thalamus: postcentral | 1.461 | .243 |
|  | Right thalamus: precentral | 3.013 | .060 |
|  | Right thalamus: temporal | 8.842 | .001 |
|  | Right thalamus: global | - | - |

***Group-wise spatial topology of thalamocortical segments in MNI space***


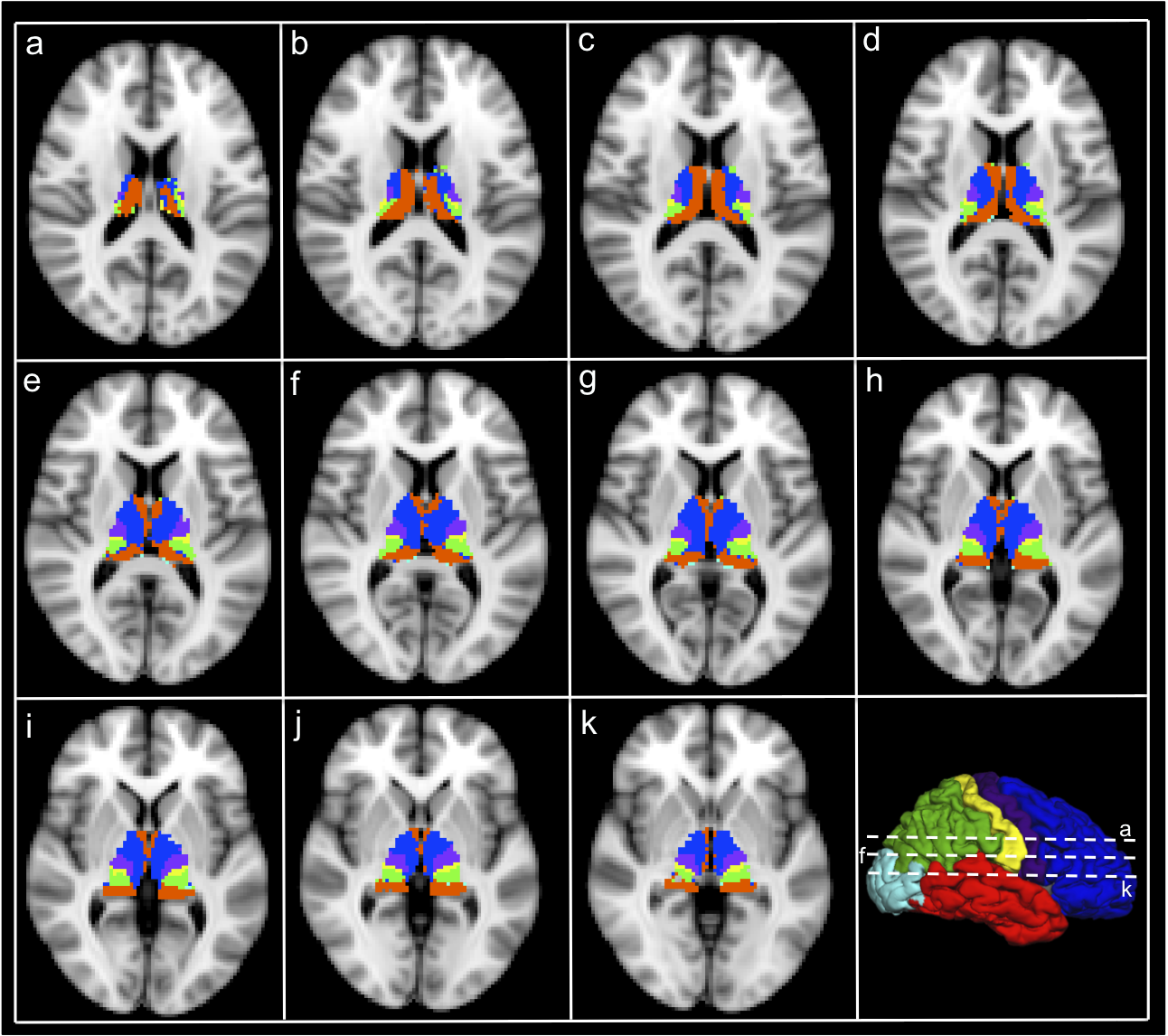


**Supplementary Figure 2.** Thalamocortical segments for all subjects in MNI space.

Superior (a) to inferior (k) axial sections through the thalamus are indicated. The colour-coded rendering of the cerebral hemisphere in the bottom right corner indicates (i) the different cortical targets and (ii) the approximate corresponding superior-most (a), middle (f) and inferior-most (k) axial sections. Thalamocortical segments: red = temporal cortex, blue = prefrontal cortex, purple = precentral cortex, yellow = postcentral cortex, green = parietal cortex, turquoise = occipital cortex. Whilst not shown here, group-wise profiles for patients were visually similar (given non-linear normalisation of all subjects to a common template). For both patients and controls, preferential temporal lobe connectivity was observed in dorsomedial and posterior thalamic regions, and predominant prefrontal connectivity was observed throughout the superior-inferior extent and preferentially including anterior and middle thalamus. Precentral, postcentral and parietal segments were spatially ordered in an anterior-to-posterior manner, respectively, located lateral to dorsomedial prefrontal segments, posterior to ventral prefrontal segments, and anterior to caudal temporal segments. There was only a small expression of occipital segments in the posterior-most aspect of the thalamus.

**References**

1. Jones DK, Williams SC, Gasston D, et al. Isotropic resolution diffusion tensor imaging with whole brain acquisition in a clinically acceptable time. Hum Brain Mapp. 2002 Apr;15:216-30.

2. Deoni SC. High-resolution T1 mapping of the brain at 3T with driven equilibrium single pulse observation of T1 with high-speed incorporation of RF field inhomogeneities (DESPOT1-HIFI). J Magn Reson Imaging. 2007 Oct;26:1106-11.

3. Deoni SC. Transverse relaxation time (T2) mapping in the brain with off-resonance correction using phase-cycled steady-state free precession imaging. J Magn Reson Imaging. 2009 Aug;30:411-7.

4. Dale AM, Fischl B, Sereno MI. Cortical surface-based analysis. I. Segmentation and surface reconstruction. Neuroimage. 1999 Feb;9:179-94.

5. Fischl B, Salat DH, Busa E, et al. Whole brain segmentation: automated labeling of neuroanatomical structures in the human brain. Neuron. 2002 Jan 31;33:341-55.

6. Desikan RS, Segonne F, Fischl B, et al. An automated labeling system for subdividing the human cerebral cortex on MRI scans into gyral based regions of interest. Neuroimage. 2006 Jul 1;31:968-80.

7. Fischl B, Stevens AA, Rajendran N, et al. Predicting the location of entorhinal cortex from MRI. Neuroimage. 2009 Aug 1;47:8-17.

8. Behrens TE, Johansen-Berg H, Woolrich MW, et al. Non-invasive mapping of connections between human thalamus and cortex using diffusion imaging. Nat Neurosci. 2003 Jul;6:750-7.

9. Traynor C, Heckemann RA, Hammers A, et al. Reproducibility of thalamic segmentation based on probabilistic tractography. Neuroimage. 2010 Aug 1;52:69-85.

10. Fischl B, Dale AM. Measuring the thickness of the human cerebral cortex from magnetic resonance images. Proceedings of the National Academy of Sciences of the United States of America. 2000 Sep 26;97:11050-5.
